# Quantitative tissue-specific dynamics of in vivo GILZ mRNA expression and regulation by endogenous and exogenous glucocorticoids

**DOI:** 10.14814/phy2.12382

**Published:** 2015-06-08

**Authors:** Vivaswath S Ayyar, Richard R Almon, William J Jusko, Debra C DuBois

**Affiliations:** 1 Department of Pharmaceutical Sciences, State University of New York at BuffaloBuffalo, New York; 2 Department of Biological Sciences, State University of New York at BuffaloBuffalo, New York; 3 New York State Center of Excellence in Bioinformatics and Life SciencesBuffalo, New York

**Keywords:** Corticosteroids, GILZ, glucocorticoids, qRT-PCR

## Abstract

Glucocorticoids (GC) are steroid hormones, which regulate metabolism and immune function. Synthetic GCs, or corticosteroids (CS), have appreciable clinical utility via their ability to suppress inflammation in immune-mediated diseases like asthma and rheumatoid arthritis. Recent work has provided insight to novel GC-induced genes that mediate their anti-inflammatory effects, including glucocorticoid-induced leucine zipper (GILZ). Since GILZ comprises an important part of GC action, its regulation by both drug and hormone will influence CS therapy. In addition, GILZ expression is often employed as a biomarker of GC action, which requires judicious selection of sampling time. Understanding the in vivo regulation of GILZ mRNA expression over time will provide insight into both the physiological regulation of GILZ by endogenous GC and the dynamics of its enhancement by CS. A highly quantitative qRT-PCR assay was developed for measuring GILZ mRNA expression in tissues obtained from normal and CS-treated rats. This assay was applied to measure GILZ mRNA expression in eight tissues; to determine its endogenous regulation over time; and to characterize its dynamics in adipose tissue, muscle, and liver following treatment with CS. We demonstrate that GILZ mRNA is expressed in several tissues. GILZ mRNA expression in adipose tissue displayed a robust circadian rhythm that was entrained with the circadian oscillation of endogenous corticosterone; and is strongly enhanced by acute and chronic dosing. Single dosing also enhanced GILZ mRNA in muscle and liver, but the dynamics varied. In conclusion, GILZ is widely expressed in the rat and highly regulated by endogenous and exogenous GCs.

## Introduction

Glucocorticoids (GCs) are a class of pleiotropic steroid hormones extensively involved in regulating development, metabolism, and immune function. They are produced endogenously in the adrenal cortex and regulated by the HPA axis in a negative-feedback fashion. Input from the suprachiasmatic nucleus imparts circadian rhythmicity to the HPA axis – causing a distinct circadian pattern of GC release. GCs have wide-ranging physiological effects owing to the ubiquitous distribution of the glucocorticoid receptor (GR) across most tissues in the body. They play a major role in regulating the production, storage, use, and distribution of substrates for systemic energy metabolism (Dallman et al. 1993). In addition, GCs possess potent immune suppressive effects that are clinically exploited through the pharmacologic use of corticosteroids (CS) for anti-inflammatory therapy.

The physiological and pharmacological effects of GCs are complex and involve extensive changes in gene expression in multiple tissues (Almon et al. 2005a,b, 2007b). Mechanistically, hormone-bound GR translocates to the nucleus where it binds to specific DNA sequences known as GREs in the 5′-upstream promoter or enhancer regions of target genes, leading to transcriptional changes by altering chromatin structure (Newton 2000; Barnes 2006). These interactions of the ligand bound GR positively or negatively regulates the expression of most metabolic and some immune-related genes; a process generally referred to as “transactivation”. Alternatively, “transrepression” involves physical interaction between ligand-activated GR and other transcription factors such as NF*κ*B and AP-1, which indirectly modulates expression of their target genes and subsequent immunomodulation (Barnes 1998; Adcock and Caramori 2001). Classical dogma contends that the anti-inflammatory effects of GCs are mediated predominantly by transrepression, while the undesired metabolic side effects primarily stem from transactivation (Adcock and Caramori 2001; Barnes 2006). However, this view has been challenged in recent years (Newton and Holden 2007; Vandevyver et al. 2013).

Glucocorticoid-induced leucine zipper (GILZ) is a gene that is highly regulated by both endogenous and exogenous GCs via transactivation, and is often used as a marker for GC action (Hinds et al. 2010; Sarabdjitsingh et al. 2010; Broccoletti et al. 2011; Schmidt et al. 2013; Whirledge and Cidlowski 2013). GILZ was first identified in 1997 in a study isolating mRNA transcriptionally enhanced in dexamethasone (DEX)-treated thymocytes (D'Adamio et al. 1997). The GILZ protein is proposed to be an important mediator of anti-inflammatory GC action (Ayroldi 2001; Fan and Morand 2012). GILZ protein mediates its anti-inflammatory effects in a manner similar to GCs itself – through interactions with AP-1 and NF*κ*B pathways (Mittelstadt and Ashwell 2001; Di Marco et al. 2007).

The majority of current data characterizing GILZ mRNA expression has been performed in vitro, while the available in vivo reports utilize single or sparse sampling times. Additionally, no data published thus far examines the in vivo GILZ expression profile versus time in normal animals (which should exhibit time of day fluctuations due to the circadian output of endogenous GCs) or as a function of CS treatment. Therefore, a quantitative approach to characterize GILZ mRNA expression using a rich in vivo time-series will provide more insight to not only the physiological regulation of GILZ but also the pharmacodynamics of GILZ enhancement by CS.

In this report, we describe a TAQMAN-based qRT-PCR assay developed for the highly quantitative measurement of GILZ mRNA. The assay was successfully applied to demonstrate that GILZ mRNA is expressed ubiquitously across tissues in the rat and that it follows a robust circadian rhythm in adipose tissue that is entrained to corticosterone (CST) production. Further, dynamics of GILZ mRNA expression was measured following a single dose of methylprednisolone (MPL), a corticosteroid of intermediate potency, in rat adipose, muscle, and liver tissue. Additionally, GILZ mRNA dynamics was assessed in adipose tissue from animals that received chronic infusion of MPL.

## Materials and Methods

### Animal experimentation

Tissue samples used to characterize expression of GILZ mRNA in normal rats and rats given a single bolus of MPL were obtained from population-based animal studies previously conducted in our laboratory. An extensive description of these studies can be found in our previously published reports (Hazra et al. 2007; Almon et al. 2008a). Brief descriptions of the two animal studies are provided here. An additional study involving chronic administration was also performed and is detailed below. All protocols adhered to the ‘Principles of Laboratory Animal Care’ (NIH publication 85–23, revised in 1985) and were approved by the University at Buffalo Institutional Animal Care and Use Committee. In all three studies, animals were acclimated for approximately 2 weeks prior to experimentation. Animals were housed in a dedicated room under a 12 h:12 h light:dark cycle and were subjected to minimal environmental disturbance. Animals were sacrificed by exsanguination from the abdominal aorta under ketamine/xylazine anesthesia (80 and 10 mg/kg), and plasma harvested using EDTA as anticoagulant (4 mmol/L final concentration). Tissues harvested were rapidly dissected, frozen in liquid nitrogen, and stored at −80°C until RNA preparation. Animals sacrificed at the same time point in a particular study were treated as triplicate measurements.

### Circadian rhythm study

Fifty-four normal male Wistar rats purchased from Harlan Laboratories (Indianapolis, IN) were housed and allowed to acclimatize in a constant-temperature environment (22°C) equipped with a 12-h light/dark cycle. Animals were sacrificed by exsanguination on three successive days at 18 different time points.

### Single bolus MPL study

Sixty normal male Wistar rats purchased from Harlan Laboratories were utilized. Each animal was given one dose of methylprednisolone sodium succinate (Solu-Medrol; Upjohn, Kalamazoo, MI) (50 mg/kg) via intramuscular injection between 1.5 and 3 h after lights on. Animals were sacrificed at 18 different time points after receiving MPL injection. Control animals were sacrificed in triplicate at 12 and 24 h after saline injection.

### Chronic MPL-infusion study

Thirty-nine normal male Wistar rats (325–349 g) were purchased from Harlan Sprague Dawley Inc. (Indianapolis, IN). The treatment group (*n* = 27) was given 0.3 mg/kg/h of methylprednisolone sodium succinate (Solu-Medrol; Upjohn) reconstituted with supplied vehicle. Alzet osmotic mini-pumps (Model 2001; Alza, Palo Alto, CA) with a flow rate of 1 *μ*L/h were subcutaneously implanted 1.5–3.5 h after lights on corresponding to the nadir of the endogenous CST rhythm. For each rat, the concentration of the pump solution was prepared based on the predose body weight of the rat. The pumps were equilibrated overnight at 37°C in saline prior to implantation in order to ensure a constant zero-order release rate. Animals in the control group (*n* = 12) were implanted with vehicle filled pumps. Animals were sacrificed by exsanguination over 7 days at 6, 9, 12, 18, and 24 h and 2, 3, 4, and 7 days following pump implantation (*n* = 3 animals per time point). Vehicle controls were sacrificed at 6, 12, 18, and 24 h postpump implantation.

### Plasma steroid assays

Plasma CST and MPL concentrations were determined by a sensitive normal-phase high-performance liquid chromatography (HPLC) method as previously described (Ebling et al. 1985; Sun et al. 1998). The limit of quantitation was 10 ng/mL. The interday and intraday coefficients of variation (CV) were less than 10%.

### Tissue RNA preparation

Frozen tissue samples were transferred from −80°C to a mortar and ground into a fine powder under liquid nitrogen. Tissues were weighed and added to prechilled TRI Reagent (Invitrogen, Carlsbad, CA) and homogenized. Total RNA extractions were carried out using TRI Reagent and further purified by passage through RNeasy mini-columns (QIAGEN, Valencia, CA) according to the manufacturer's protocols for RNA clean-up. Final RNA preparations were eluted in RNase-free water and stored in RNase-free tubes at −80°C. RNA concentrations were quantified spectrophotometrically (NanoDrop 2000c; Thermo Scientific, Waltham, MA), and purity and integrity were assessed by formaldehyde agarose gel electrophoresis. All samples exhibited 260/280 absorbance ratios of approximately 2.0, and all showed intact ribosomal 28S and 18S RNA bands in an approximate ratio of 2:1 as visualized by ethidium bromide staining.

### qRT-PCR assay development

A GILZ-specific qRT-PCR assay was developed and validated according to MIQE standards (Bustin et al. 2009). Quantitative RT-PCR involved use of an in vitro transcribed GILZ cRNA standard, GILZ-specific TAQMAN-based probe, and a single-step assay with GILZ mRNA normalized to total RNA in the assay. The cRNA standard was constructed as follows: A region of 1040 base pairs (bp), which covered the entire gene coding sequence (404 bp), and did not share homology with other genes was selected using rat GILZ RefSeq from NCBI (NM_031345 positions 40 to 1079). This sequence was cloned into pCR 2.1 TOPO vector (Invitrogen) using conventional RT-PCR procedures according to the manufacturer's directions. Automated Sanger sequencing of cloned plasmid DNA for verification was performed at the Roswell Park Cancer Institute DNA Facility. Linearized plasmid was in vitro transcribed using Megascript T7 kits (Ambion, Austin TX) according to the manufacturer's instructions. Purified cRNA was quantified spectrophotometrically, and purity and integrity assessed by formaldehyde agarose gel electrophoresis.

### qRT-PCR conditions

Primer sets and probes were designed using RealTime Design software (Biosearch Technologies Inc., Novato, CA) and were custom synthesized by Biosearch Technologies. Preliminary experiments were conducted to maximize probe, primer, and MgCl_2_ concentrations. Primer and probe sequences and assay conditions are presented in Table1. Assays employed Stratagene Brilliant II RT-PCR One Step Core Reagent kits (Agilent Technologies, Cedar Creek, TX). The qRT-PCR was performed in a Stratagene MX3005P fluorescence-based thermocycler. Final assay volumes were 25 *μ*L, and included 2.5 *μ*L total RNA at a concentration of 25 ng/*μ*L (final RNA concentration of 62.5 ng). A standard curve generated with the cRNA standards consisting of seven concentrations (23.88–2292 femtograms/tube) was included in each experiment. *R*^2^ values were in all cases greater than 0.988. Efficiencies of all runs were greater than 90%. Samples were run in triplicate; intra- and interassay variabilities were less than 20% in all cases. Additional reverse transcriptase (RT) minus controls were run for each RNA sample to confirm absence of genomic DNA contamination; all samples exhibited lack of amplification in RT minus controls. Additionally, a quality control consisting of a single rat liver RNA sample was included in every qRT-PCR run to ensure equivalency of individual experiments.

**Table 1:** qRT-PCR probe and primer sequences and assay conditions

Description	Sequence	Concentration
Forward primer	5′- GGAGGTCCTAAAGGAGCAGATTC- 3′	150 nmol/L
Reverse primer	5′- GCGTCTTCAGGAGGGTATTCTC- 3′	300 nmol/L
Probe	5′ FAM-TGAGCTGGTTGAGAAGAACTCGCA-BHQ-1 3′	100 nmol/L
MgCl_2_	–	3 mmol/L

### Data analysis

Plasma CST data and GILZ mRNA circadian data were fitted to harmonic functions using FOURPHARM software (Krzyzanski et al. 2000). Area under the effect curve values (AUEC) were calculated from GILZ mRNA expression data using Phoenix WinNonlin 6.3 (Pharsight, Mountain View, CA).

## Results

### GILZ mRNA expression in normal animals

**Figure 1:**
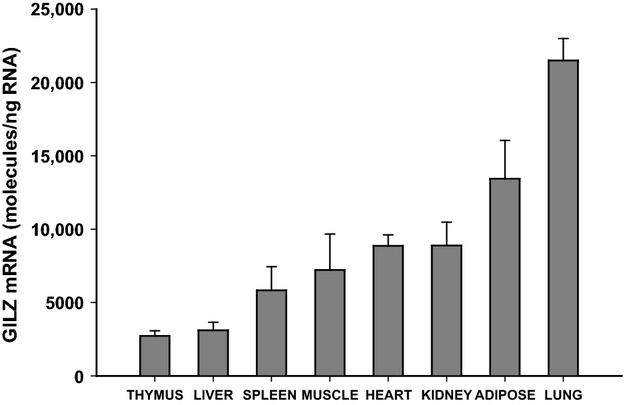
GILZ mRNA expression in various rat tissues from nondrug-treated animals. Bars represent means and error bars are one standard deviation (*n* = 3).

Expression levels of GILZ were measured in liver, spleen, thymus, lung, skeletal muscle (gastrocnemius), kidney, heart, and adipose tissue from normal male Wistar rats. Tissues employed were obtained from nondrug-treated animals from our previously conducted time-course study (Hazra et al. 2007). GILZ mRNA levels were measured in tissues harvested from animals sacrificed during the early dark phase of a tightly controlled 12 h:12 h light:dark cycle. Figure1 shows GILZ mRNA expression in various rat tissues. GILZ was expressed across all tissues measured, with maximal expression in lung (21495 ± 1499 molecules/ng RNA) and least expression in thymus (2714 ± 359 molecules/ng RNA). The varying levels of expression observed between adipose, muscle, and liver prompted us to further investigate GILZ mRNA dynamics in those tissues.

### Circadian regulation of GILZ mRNA

**Figure 2:**
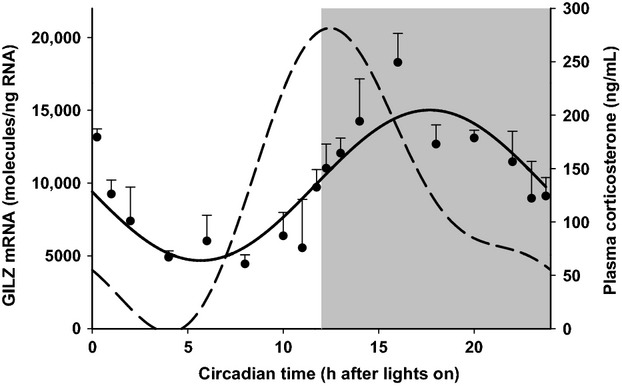
GILZ mRNA in rat adipose tissue as a function of circadian time. Closed circles represent mean and error bars one standard deviation (*n* = 3). The solid line depicts the fitting of GILZ mRNA data. The dashed line represents plasma CST measurements from the same animals. Unshaded regions depict light phase and shaded regions depict dark phase of the 24 h cycle.

Endogenous cyclic regulation of GILZ mRNA expression was assessed in adipose tissue obtained from normal animals housed under a tightly controlled 12-h light/dark cycles. Three animals were sacrificed at each of 18 different time points across the 24 h cycle. Figure2 displays the GILZ mRNA expression profile in adipose tissue, which exhibits a robust circadian rhythm. GILZ mRNA levels peak in the dark period (18867 ± 2347 molecules/ng RNA) at 16 h after lights on (corresponding to 4 h after lights off) and shows a trough in the light period (4446 ± 622 molecules/ng RNA) at 8 h after lights on. Superimposed on this figure is the profile of corticosterone (the endogenous GC in the rat) measured by HPLC in plasma samples taken from these same animals. These data indicate that GILZ mRNA regulation in vivo follows a pattern that is entrained to that of the corticosterone rhythm.

### Dynamics of GILZ mRNA following MPL dosing

**Figure 3:**
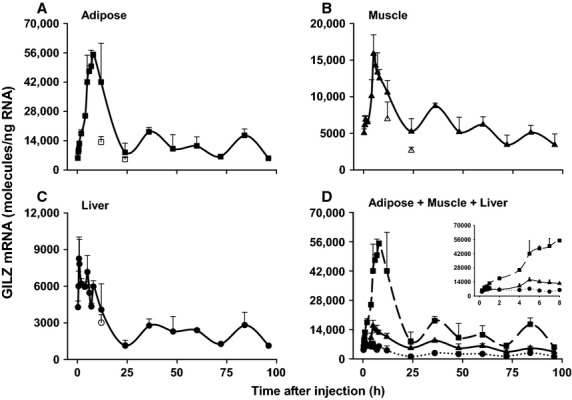
Dynamics of GILZ mRNA regulation by acute MPL bolus in rat adipose (A), muscle (B), and liver (C). Panel D provides direct comparison of GILZ mRNA expression profiles in all three tissues. The inset in Panel (D) presents an expanded version of the data through 8 h. Closed boxes, triangles, and circles are means of treated animals in adipose, muscle, and liver; open boxes, triangles, and circles are means of light/dark control animals in adipose, muscle, and liver. Error bars represent one standard deviation (*n* = 3). Note: time in the plot represents time following MPL dosing at 2 h after lights on.

In addition to regulation by endogenous GC, previous reports document GILZ mRNA upregulation by CS (Ayroldi and Riccardi 2009; Beaulieu and Morand 2011). GILZ mRNA expression was measured in RNA extracted from rat adipose tissue, liver, and muscle. Animals were given a single 50 mg/kg dose of MPL and sacrificed at various times after injection (15 min to 96 h; *n* = 3 animals per time point). MPL pharmacokinetics in these animals was published previously (Hazra et al. 2007); plasma drug concentrations decline biexponentially and are undetectable by 8 h postdosing. Figure3 describes the dynamics of GILZ mRNA expression as a function of time in rat adipose, muscle, and liver. In addition to differences in peak GILZ expression, times to peak expression also varied between the tissues analyzed.

Figure3A demonstrates that GILZ mRNA expression is strongly upregulated upon dosing with MPL in adipose tissue; it shows a steady increase in expression starting as early as 30 min after injection and reaches peak levels (55,053 ± 1342 molecules/ng RNA) around 8 h. Figure3B shows that GILZ mRNA is also upregulated in muscle, but to a lesser extent. GILZ expression is enhanced 30 min postdosing and sharply rises to peak levels (15,851 ± 2598 molecules/ng RNA) by 5 h. Figure3C depicts upregulation of GILZ mRNA in liver by MPL. While expression is evidently enhanced in liver, peak levels (8262 ± 1763 molecules/ng RNA) are reached as early as 45 min after dosing. Figure3D directly compares the time profiles of GILZ mRNA expression in all three tissues. Integration of these curves demonstrates that the total GILZ mRNA expression in these three tissues differ substantially in magnitude. The AUEC_0–24 h_ values for GILZ mRNA expression were 742,546 molecules/ng RNA.h in adipose, 224,136 molecules/ng RNA.h in muscle and 99,036 molecules/ng RNA.h in liver.

### Re-establishment of GILZ circadian rhythm post-MPL dosing

**Figure 4:**
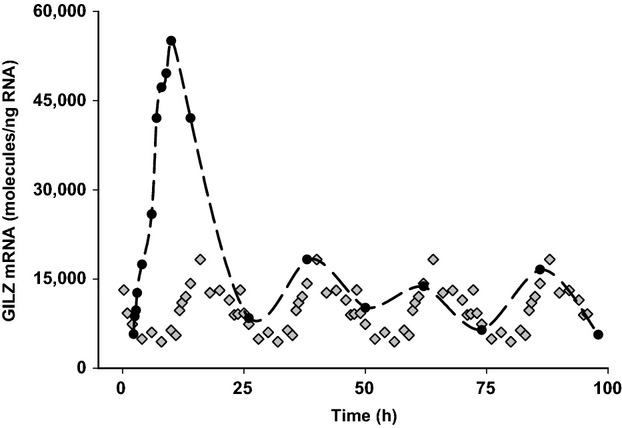
Reestablishment of GILZ circadian rhythm postacute dosing. Closed circles are means of MPL-dosed animals and shaded triangles are means of control animals. Note: time in the plot reflects time after lights on.

Since steady-state expression of GILZ mRNA shows a circadian pattern, enhanced expression by exogenous CS must be interpreted within the context of this circadian pattern. Figure4 presents an integrated depiction of circadian and MPL-enhanced GILZ mRNA expression in adipose tissue. The response of MPL clearly exceeds normal circadian variation**.** Following perturbation by MPL, GILZ mRNA expression eventually reestablishes its circadian rhythm. The baseline AUEC from time 0 to 24 h for adipose GILZ mRNA expression in normal animals was estimated to be 232,369 molecules/ng RNA.h, while AUEC_0–24 h_ for GILZ mRNA in animals dosed with MPL bolus was 742,546 molecules/ng RNA.h. Therefore, the enhancement by MPL was 510,177 molecules/ng RNA.h.

### Dynamics of GILZ mRNA regulation by MPL infusion

**Figure 5:**
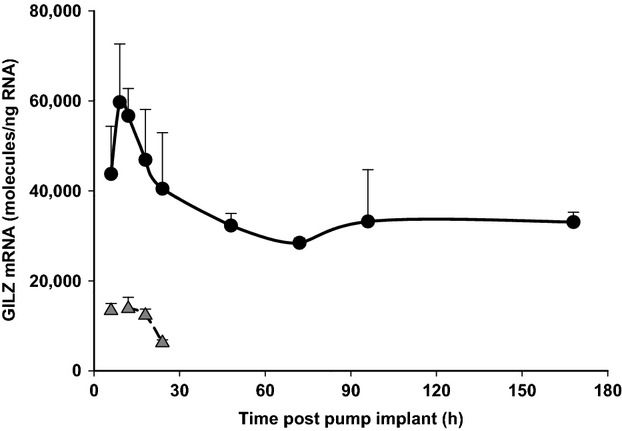
Dynamics of GILZ mRNA regulation by chronic MPL infusion in rat adipose tissue. Closed circles are means of treated animals, shaded triangles are means of vehicle-infused controls, and error bars one standard deviation (*n* = 3).

GILZ mRNA expression was analyzed in adipose obtained from animals subcutaneously infused with MPL (6 to 168 h; *n* = 3 animals per time point). As shown in Fig.5, MPL infusion produced a robust increase in GILZ mRNA expression with the peak levels reaching 59,720 ± 12,947 molecules/ng RNA at around 9 h. However, a new steady-state baseline was reached with the constant presence of drug. Therefore, GILZ mRNA expression levels remained significantly higher than in normal animals through all time points measured. Vehicle-infused control animals showed GILZ mRNA levels similar to normal animals except for the 6 h group; possibly due to higher corticosterone concentrations postsurgery.

## Discussion

As its name suggests, GILZ is a glucocorticoid-inducible gene that encodes a leucine zipper protein, and is also known as Tsc22d3 due to the significant homology that its protein shares with the Tsc22 leucine zipper family. The coding sequence of human GILZ shares significant homology with murine GILZ; specifically, 97% with mouse GILZ and 91% with rat GILZ. Here, we demonstrate that varying levels of GILZ mRNA expression are seen across several tissues. We have characterized its physiological regulation by endogenous and exogenous GC in vivo. The GILZ gene is located on the X-chromosome, with six GREs in its promoter, three of which are functionally active (Ayroldi and Riccardi 2009; Pinheiro et al. 2013). Transcriptional regulation of GILZ by GC involves the GC/GR complex binding to these multiple GRE sequences to enhance mRNA synthesis. This mechanism conceivably explains the sensitive and substantial effects of GC on GILZ expression. Over the past decade, GILZ has emerged as a novel mediator of the anti-inflammatory effects of CS. Through direct protein–protein interactions with transcription factors and signaling molecules, GILZ can block the transcription of their downstream proinflammatory targets such as TNF-*α*, IL-1*β,* and IL-6 (Mittelstadt and Ashwell 2001; Fan and Morand 2012; Hahn et al. 2014). Therefore, understanding the physiology of GILZ expression in vivo and its regulation by GC are of clinical importance. Furthermore, GILZ mRNA expression can be used as a marker for in vivo GC action.

The expression of GILZ at both the mRNA and protein levels in various human cell lines and some tissues was previously documented (Cannarile et al. 2001; Berrebi et al. 2003; Eddleston et al. 2007; Aguilar et al. 2013). Here, we demonstrate constitutive GILZ expression in all tissues examined. Results from our tissue panel (Fig.1) are consistent with GILZ expression data from prior studies. In particular, GILZ mRNA expression across tissues showed remarkable consistency between our results and relative expression in human tissues measured by Northern hybridization as reported by Cannarile et al. (2001). The GILZ mRNA was expressed the least in thymus and the highest in lung, the latter expressing eight to ninefold higher levels than thymus. Furthermore, Cannarile et al. (2001) demonstrated complete concordance between GILZ mRNA and protein expression, which suggests that GILZ is regulated at the transcriptional rather than posttranscriptional level. One limitation of using whole tissue is cell heterogeneity in the samples. Typical lymphoid tissues such as thymus and spleen are rich in immune cells, while adipose and lung are complex organs made of not only tissue-specific cell types but also house a variety of immune cells. Interestingly, the richness of immune cells in various tissues did not seem to correlate with the amount of GILZ expressed. Furthermore, expression of GILZ was observed in several cell types in vitro such as hepatocytes, epithelial cells, osteocytes, cardiomyocytes, and skeletal muscle cells from both murine and human models (D'Adamio et al. 1997; Eddleston et al. 2007; Hamdi et al. 2007; Beaulieu et al. 2010; Bruscoli et al. 2010; He et al. 2012). Together, this suggests that GILZ is expressed in nonlymphoid tissues as well.

Enhanced expression of GILZ by GC is well documented, albeit primarily in in vitro studies, and GILZ upregulation has been suggested to contribute to the immunosuppressive effects of GC. Initial work suggested that GILZ expression was restricted to DEX-treated lymphoid tissues (D'Adamio et al. 1997). GILZ upregulation was seen in DEX-treated murine T-cells derived from spleen and lymph nodes and in human mast cells (D'Adamio et al. 1997; Godot et al. 2006). GILZ expression increased in a dose-dependent fashion in splenocytes and peritoneal macrophages in rats postrestraint-stress or upon treatment with DEX, which led to reduced inflammatory responses to LPS as demonstrated by decreased levels of IL-1, IL-6, IL-12, and TNF (Wang et al. 2008, 2012). In addition, Kupffer cells were found to be the predominant source of GILZ in liver in both normal human samples and patients with alcoholic hepatitis (AH). It has been proposed that GILZ upregulation in patients with AH may contribute to the beneficial effect of GC by reducing liver inflammation (Hamdi et al. 2007). Dendritic cells (DC) play a critical role in the adaptive immune response through activation of T-lymphocytes, by virtue of their antigen-presenting ability in association with MHC molecules. GILZ is expressed in human DC where it mediates the immunosuppressive effects of GC by inhibiting DC maturation, antigen presentation, and subsequently its ability to stimulate T-lymphocytes (Cohen et al. 2006).

Limited studies also exist showing GC-regulated GILZ expression in nonlymphoid cells, including cardiac and skeletal muscle as well as epithelium. GILZ is expressed in human airway epithelial cells where they significantly inhibited IL-1*β* and LPS-induced activation of NF*κ*B (Eddleston et al. 2007). GC-enhanced GILZ expression in mouse heart and in primary cultured rat cardiomyocytes, even at doses as low as 0.1 *μ*mol/L-DEX (Aguilar et al. 2013). Furthermore, GILZ mediated the anti-myogenic effect of GC in myoblasts by inhibition of myoblast differentiation and their maturation into myotubes (Bruscoli et al. 2010). Although GILZ expression was previously found in kidney (D'Adamio et al. 1997), evidence of its physiological function there was established following observation that aldosterone upregulates GILZ expression in cell lines derived from mouse kidney collecting duct principal cells. GILZ induction by aldosterone strongly stimulated epithelial sodium channel-mediated Na^+^ transport by inhibiting ERK signaling (Soundararajan et al. 2005). There are no prior studies of GILZ expression in mature adipose tissue. However, Shi et al. have shown that GILZ antagonizes adipocyte differentiation in mesenchymal cells by acting as a sequence-specific transcriptional repressor by binding in the promoter region of the gene encoding peroxisome-proliferator-activated receptor-*γ*2 (PPAR-*γ*2), the same receptor which GC bind to induce adipogenesis (Shi et al. 2003; Gimble et al. 2009).

Since the majority of work on GC-mediated GILZ regulation has been carried out in cell culture, little information is available in the literature on the dynamics of such regulation in vivo. An initial question we addressed was whether the magnitude of GILZ expression varied with time of day in a normal animal. Circadian rhythmicity in the expression of a large number of genes has been demonstrated by us in several tissues (Sukumaran et al. 2010). Since endogenous GC are released in a rhythmic fashion, it can be expected that its downstream target genes in peripheral tissues may follow similar fluctuations over time. Indeed, many GC-regulated genes, including tyrosine aminotransferase (TAT), glutamine synthetase, cholesterol-7a-hydroxylase, and the glucocorticoid receptor itself exhibit circadian rhythms, as documented by us and others (Van Cantfort and Gielen 1979; Angelova and Angelov 1998; Yao et al. 2006; Almon et al. 2008b). Such rhythmic variations in gene expression can influence drug action, including pharmacodynamic responses, due the availability or functioning of target proteins. Hence, conclusions made from rat studies may be distorted without considering these rhythmic oscillations (Yao et al. 2006). Here, we demonstrate that GILZ mRNA expression follows a distinct circadian rhythm of expression in rat adipose tissue (Fig.2). It can be observed that in vivo GILZ mRNA expression shows a rhythm that is entrained to endogenous CST production. Based on our data fitting in Fig.2, there is an evident time delay between CST production and GILZ mRNA synthesis; plasma CST levels peak just after the beginning of the dark period, while GILZ mRNA reaches maximal levels by 5 h into the 12 h dark period. It can be observed that there is a delay between the fitted and experimental peaks, the latter occurring around 4 h into the dark period. It is important to note while evaluating circadian data that nocturnal animals such as rats possess some circadian gene expression cycles that are essentially reversed in humans, who are mostly diurnal in nature.

GILZ mRNA dynamics was analyzed in rat adipose, liver, and muscle harvested at various times following perturbation by a single dose of MPL (Fig.3). Our results indicate that while GILZ expression was clearly enhanced by MPL in all three tissues, maximal response (*R*_max_) and time of peak response (*T*_max_) varied. Furthermore, AUEC_0–24 h_ for adipose GILZ mRNA expression was 7.5-fold and 3.3-fold higher than that of liver and muscle. AUEC_0–24 h_ for GILZ mRNA expression in muscle was about 2.5-fold higher than in liver. When comparing the circadian production of adipose GILZ mRNA to its dynamics upon MPL dosing in the same tissue (Fig.4), GILZ mRNA expression is sharply enhanced by MPL from the early points until around 8 h postdosing, when compared to baseline levels. However, from 24 h through the last time point (96 h), GILZ expression reestablishes its baseline circadian rhythm (Fig.4). While the AUEC of GILZ enhancement by MPL above baseline was calculated in dosed animals, one limitation is that we assume a normal CST rhythm in these animals. In reality, CS such as DEX and MPL cause suppression of endogenous CST by binding to GR in the pituitary, thereby modulating its release from the adrenal cortex (Cole et al. 2000; Yao et al. 2008). This, is in turn, could modulate endogenous GILZ expression. However, it is also true that MPL binds GR with higher affinity than CST, and predominantly exerts receptor/gene-mediated effects when present in the system.

We also examined adipose GILZ mRNA dynamics after chronic infusion of MPL in rats subcutaneously infused with MPL (Fig.5). Enhancement of GILZ mRNA expression by MPL in these animals showed a profile similar to acutely dosed animals with respect to *R*_max_ and *T*_max_. However, following an initial decline, GILZ mRNA levels plateaued beyond 48 h at about 31,749 ± 2236 molecules/ng RNA, well higher than rhythmic baseline levels. Previous cluster analyses on microarray data in liver and muscle following chronic MPL infusion indicate that, while some regulated genes exhibit steady-state levels equivalent to untreated controls, others like GILZ maintain an new enhanced steady-state level (Almon et al. 2007a,b).

Significant homology (93%) is observed between GILZ mRNA in rat and mouse. Soundararajan et al. (2007) have reported the existence of at least two distinct isoforms in mouse; transcript variant 1 (NM_001077364.1) and transcript variant 2 (NM_010286.4). Alignment of our qRT-PCR amplicon sequence to both transcript variants in mouse revealed that the assay would measure both splice variants, assuming that similar splice variants also exist in rat. Therefore, total measured GILZ mRNA expression would include both variants.

Animal experiments are often conducted to investigate time-sensitive phenomena in vivo. It is well established that GC signaling is highly dynamic with molecular to phenotypic time scales from seconds to years (Dickmeis et al. 2013). However, it is interesting to note that full recovery of some genes to baseline levels may take days following perturbation by CS. Therefore, times of sampling over the 96-h period in the acute MPL study were chosen based on previous experiments indicating that the effect of the drug was most significant at the early times following dosing, but full recovery in some cases required as long as 96 h (Almon et al. 2008a). CS and their molecular actions in vivo are being studied extensively. However, two subtle yet important elements that are often overlooked in these studies are the circadian input of endogenous GC and the extended time-course over which the receptor/gene-mediated mechanisms of CS effects take place. In this study, through the development and use of a highly quantitative qRT-PCR assay for the measurement of GILZ mRNA expression in vivo, we demonstrated the significance of the endogenous GC circadian rhythm on GILZ gene expression and have characterized the physiological regulation of GILZ mRNA expression by MPL in rat adipose, muscle, and liver.

In conclusion, this study demonstrates that GILZ mRNA shows ubiquitous expression across several tissues in vivo, that it follows a robust circadian rhythm of expression in rat adipose tissue, and that its expression is strongly enhanced by MPL in adipose, muscle, and liver, but with varying dynamics. Gaining an understanding into the physiological regulation of GILZ by endogenous and exogenous GC may provide insight into therapeutic strategies to upregulate its expression to suppress inflammation. Involvement of GILZ has been reported in disorders such as rheumatoid arthritis and osteoporosis (Shi et al. 2007; Beaulieu et al. 2010; Beaulieu and Morand 2011), but much remains to be explored regarding the regulation of GILZ expression by these diseases. Another interesting and unexplored area in GILZ research is understanding its physiological relevance in mature adipose tissue. While GILZ mRNA expression dynamics were studied in tissues that are heavily involved in metabolic regulation, it is possible that physiological and pharmacological regulation of GILZ might vary in structurally and/or immunologically related organs such as lung and bone.

## References

[b1] Adcock IM, Caramori G (2001). Cross-talk between pro-inflammatory transcription factors and glucocorticoids. Immunol. Cell Biol.

[b2] Aguilar DC, Strom J, Xu B, Kappeler K, Chen QM (2013). Expression of glucocorticoid-induced leucine zipper (GILZ) in cardiomyocytes. Cardiovasc. Toxicol.

[b3] Almon RR, Dubois DC, Jin JY, Jusko WJ (2005a). Pharmacogenomic responses of rat liver to methylprednisolone: an approach to mining a rich microarray time series. AAPS J.

[b4] Almon RR, Lai W, DuBois DC, Jusko WJ (2005b). Corticosteroid-regulated genes in rat kidney: mining time series array data. Am. J. Physiol. Endocrinol. Metab.

[b5] Almon RR, DuBois DC, Jusko WJ (2007a). A microarray analysis of the temporal response of liver to methylprednisolone: a comparative analysis of two dosing regimens. Endocrinology.

[b6] Almon RR, DuBois DC, Yao Z, Hoffman EP, Ghimbovschi S, Jusko WJ (2007b). Microarray analysis of the temporal response of skeletal muscle to methylprednisolone: comparative analysis of two dosing regimens. Physiol. Genomics.

[b7] Almon RR, Yang E, Lai W, Androulakis IP, DuBois DC, Jusko WJ (2008a). Circadian variations in rat liver gene expression: relationships to drug actions. J. Pharmacol. Exp. Ther.

[b8] Almon RR, Yang E, Lai W, Androulakis IP, Ghimbovschi S, Hoffman EP (2008b). Relationships between circadian rhythms and modulation of gene expression by glucocorticoids in skeletal muscle. Am. J. Physiol. Regul. Integr. Comp. Physiol.

[b9] Angelova KC, Angelov CG (1998). Cosinor analysis of circadian oscillations of amino acid catabolizing enzymes in temporal pattern of nutrient input. Z. Ernahrungswiss.

[b10] Ayroldi E (2001). Modulation of T-cell activation by the glucocorticoid-induced leucine zipper factor via inhibition of nuclear factor kappaB. Blood.

[b11] Ayroldi E, Riccardi C (2009). Glucocorticoid-induced leucine zipper (GILZ): a new important mediator of glucocorticoid action. FASEB J.

[b12] Barnes PJ (1998). Anti-inflammatory actions of glucocorticoids: molecular mechanisms. Clin. Sci.

[b13] Barnes PJ (2006). How corticosteroids control inflammation: quintiles prize lecture 2005. Br. J. Pharmacol.

[b14] Beaulieu E, Morand EF (2011). Role of GILZ in immune regulation, glucocorticoid actions and rheumatoid arthritis. Nat. Rev. Rheumatol.

[b15] Beaulieu E, Ngo D, Santos L, Yang YH, Smith M, Jorgensen C (2010). Glucocorticoid-induced leucine zipper is an endogenous antiinflammatory mediator in arthritis. Arthritis Rheum.

[b16] Berrebi D, Bruscoli S, Cohen N, Foussat A, Migliorati G, Bouchet-Delbos L (2003). Synthesis of glucocorticoid-induced leucine zipper (GILZ) by macrophages: an anti-inflammatory and immunosuppressive mechanism shared by glucocorticoids and IL-10. Blood.

[b17] Broccoletti T, Del Giudice E, Cirillo E, Vigliano I, Giardino G, Ginocchio VM (2011). Efficacy of very-low-dose betamethasone on neurological symptoms in ataxia-telangiectasia. Eur. J. Neurol.

[b18] Bruscoli S, Donato V, Velardi E, Di Sante M, Migliorati G, Donato R (2010). Glucocorticoid-induced leucine zipper (GILZ) and long GILZ inhibit myogenic differentiation and mediate anti-myogenic effects of glucocorticoids. J. Biol. Chem.

[b19] Bustin SA, Benes V, Garson JA, Hellemans J, Huggett J, Kubista M (2009). The MIQE guidelines: minimum information for publication of quantitative real-time PCR experiments. Clin. Chem.

[b20] Cannarile L, Zollo O, D'Adamio F, Ayroldi E, Marchetti C, Tabilio A (2001). Cloning, chromosomal assignment and tissue distribution of human GILZ, a glucocorticoid hormone-induced gene. Cell Death Differ.

[b21] Cohen N, Mouly E, Hamdi H, Maillot MC, Pallardy M, Godot V (2006). GILZ expression in human dendritic cells redirects their maturation and prevents antigen-specific T lymphocyte response. Blood.

[b22] Cole MA, Kim PJ, Kalman BA, Spencer RL (2000). Dexamethasone suppression of corticosteroid secretion: evaluation of the site of action by receptor measures and functional studies. Psychoneuroendocrinology.

[b23] D'Adamio F, Zollo O, Moraca R, Ayroldi E, Bruscoli S, Bartoli A (1997). A new dexamethasone-induced gene of the leucine zipper family protects T lymphocytes from TCR/CD3-activated cell death. Immunity.

[b24] Dallman MF, Strack AM, Akana SF, Bradbury MJ, Hanson ES, Scribner KA (1993). Feast and famine: critical role of glucocorticoids with insulin in daily energy flow. Front. Neuroendocrinol.

[b25] Di Marco B, Massetti M, Bruscoli S, Macchiarulo A, Di Virgilio R, Velardi E (2007). Glucocorticoid-induced leucine zipper (GILZ)/NF-kappaB interaction: role of GILZ homo-dimerization and C-terminal domain. Nucleic Acids Res.

[b26] Dickmeis T, Weger BD, Weger M (2013). The circadian clock and glucocorticoids – Interactions across many time scales. Mol. Cell. Endocrinol.

[b27] Ebling WF, Szefler SJ, Jusko WJ (1985). Methylprednisolone disposition in rabbits. Analysis, prodrug conversion, reversible metabolism, and comparison with man. Drug Metab. Dispos.

[b28] Eddleston J, Herschbach J, Wagelie-Steffen AL, Christiansen SC, Zuraw BL (2007). The anti-inflammatory effect of glucocorticoids is mediated by glucocorticoid-induced leucine zipper in epithelial cells. J. Allergy Clin. Immunol.

[b29] Fan H, Morand FE (2012).

[b30] Gimble JM, Ptitsyn AA, Goh BC, Hebert T, Yu G, Wu X (2009). Delta sleep-inducing peptide and glucocorticoid-induced leucine zipper: potential links between circadian mechanisms and obesity?. Obes. Rev.

[b31] Godot V, Garcia G, Capel F, Arock M, Durand-Gasselin I, Asselin-Labat ML (2006). Dexamethasone and IL-10 stimulate glucocorticoid-induced leucine zipper synthesis by human mast cells. Allergy.

[b32] Hahn RT, Hoppstadter J, Hirschfelder K, Hachenthal N, Diesel B, Kessler SM (2014). Downregulation of the glucocorticoid-induced leucine zipper (GILZ) promotes vascular inflammation. Atherosclerosis.

[b33] Hamdi H, Bigorgne A, Naveau S, Balian A, Bouchet-Delbos L, Cassard-Doulcier AM (2007). Glucocorticoid-induced leucine zipper: a key protein in the sensitization of monocytes to lipopolysaccharide in alcoholic hepatitis. Hepatology.

[b34] Hazra A, Pyszczynski N, DuBois DC, Almon RR, Jusko WJ (2007). Modeling receptor/gene-mediated effects of corticosteroids on hepatic tyrosine aminotransferase dynamics in rats: dual regulation by endogenous and exogenous corticosteroids. J. Pharmacokinet Pharmacodyn.

[b35] He L, Yang N, Isales CM, Shi XM (2012). Glucocorticoid-induced leucine zipper (GILZ) antagonizes TNF-alpha inhibition of mesenchymal stem cell osteogenic differentiation. PLoS ONE.

[b36] Hinds TD, Ramakrishnan S, Cash HA, Stechschulte LA, Heinrich G, Najjar SM (2010). Discovery of glucocorticoid receptor-beta in mice with a role in metabolism. Mol. Endocrinol.

[b37] Krzyzanski W, Chakraborty A, Jusko WJ (2000). Algorithm for application of Fourier analysis for biorhythmic baselines of pharmacodynamic indirect response models. Chronobiol Int.

[b38] Mittelstadt PR, Ashwell JD (2001). Inhibition of AP-1 by the glucocorticoid-inducible protein GILZ. J. Biol. Chem.

[b39] Newton R (2000). Molecular mechanisms of glucocorticoid action: what is important?. Thorax.

[b40] Newton R, Holden NS (2007). Separating transrepression and transactivation: a distressing divorce for the glucocorticoid receptor?. Mol. Pharmacol.

[b41] Pinheiro I, Dejager L, Petta I, Vandevyver S, Puimege L, Mahieu T (2013). LPS resistance of SPRET/Ei mice is mediated by Gilz, encoded by the Tsc22d3 gene on the X chromosome. EMBO Mol. Med.

[b42] Sarabdjitsingh RA, Isenia S, Polman A, Mijalkovic J, Lachize S, Datson N (2010). Disrupted Corticosterone Pulsatile Patterns Attenuate Responsiveness to Glucocorticoid Signaling in Rat Brain. Endocrinology.

[b43] Schmidt U, Kaltwasser SF, Wotjak CT (2013). Biomarkers in posttraumatic stress disorder: overview and implications for future research. Dis. Markers.

[b44] Shi X, Shi W, Li Q, Song B, Wan M, Bai S (2003). A glucocorticoid-induced leucine-zipper protein, GILZ, inhibits adipogenesis of mesenchymal cells. EMBO Rep.

[b45] Shi X, Hamrick M, Isales CM (2007). Energy Balance, Myostatin, and GILZ: factors Regulating Adipocyte Differentiation in Belly and Bone. PPAR Res.

[b46] Soundararajan R, Zhang TT, Wang J, Vandewalle A, Pearce D (2005). A novel role for glucocorticoid-induced leucine zipper protein in epithelial sodium channel-mediated sodium transport. J. Biol. Chem.

[b47] Soundararajan R, Wang J, Melters D, Pearce D (2007). Differential activities of glucocorticoid-induced leucine zipper protein isoforms. J. Biol. Chem.

[b48] Sukumaran S, Almon RR, DuBois DC, Jusko WJ (2010). Circadian rhythms in gene expression: relationship to physiology, disease, drug disposition and drug action. Adv. Drug Deliv. Rev.

[b49] Sun YN, DuBois DC, Almon RR, Jusko WJ (1998). Fourth-generation model for corticosteroid pharmacodynamics: a model for methylprednisolone effects on receptor/gene-mediated glucocorticoid receptor down-regulation and tyrosine aminotransferase induction in rat liver. J. Pharmacokinet. Biopharm.

[b50] Van Cantfort J, Gielen JE (1979). Comparison of rat and mouse circadian rhythm of cholesterol-7 alpha-hydroxylase activity. J. Steroid Biochem.

[b51] Vandevyver S, Dejager L, Tuckermann J, Libert C (2013). New insights into the anti-inflammatory mechanisms of glucocorticoids: an emerging role for glucocorticoid-receptor-mediated transactivation. Endocrinology.

[b52] Wang Y, Lu Y, Yu D, Wang Y, Chen F, Yang H (2008). Enhanced Resistance of Restraint-Stressed Mice to Sepsis. J. Immunol.

[b53] Wang Y, Ma YY, Song XL, Cai HY, Chen JC, Song LN (2012). Upregulations of glucocorticoid-induced leucine zipper by hypoxia and glucocorticoid inhibit proinflammatory cytokines under hypoxic conditions in macrophages. J. Immunol.

[b54] Whirledge S, Cidlowski JA (2013). Estradiol antagonism of glucocorticoid-induced GILZ expression in human uterine epithelial cells and murine uterus. Endocrinology.

[b55] Yao Z, DuBois DC, Almon RR, Jusko WJ (2006). Modeling circadian rhythms of glucocorticoid receptor and glutamine synthetase expression in rat skeletal muscle. Pharm. Res.

[b56] Yao Z, DuBois DC, Almon RR, Jusko WJ (2008). Pharmacokinetic/pharmacodynamic modeling of corticosterone suppression and lymphocytopenia by methylprednisolone in rats. J. Pharm. Sci.

